# Adolescents’ risk perceptions on mobile phones and their base stations, their trust to authorities and incivility in using mobile phones: a cross-sectional survey on 2240 high school students in Izmir, Turkey

**DOI:** 10.1186/1476-069X-12-10

**Published:** 2013-01-25

**Authors:** Hur Hassoy, Raika Durusoy, Ali Osman Karababa

**Affiliations:** 1 Department of Public Health, Ege University School of Medicine, Bornova, 35100, Izmir, Turkey

**Keywords:** Risk perception, Adolescent, Mobile phone, Base station, Electromagnetic field

## Abstract

**Background:**

Use of mobile phones has rapidly risen among adolescents despite a lack of scientific certainty on their health risks. Risk perception is an important determinant of behavior, and studies on adolescents’ risk perceptions of mobile phones or base stations are very scarce. This study aims to evaluate high school students’ risk perceptions on mobile phones and base stations, their trust to authorities, their opinions regarding incivility while using mobile phones and to assess associated factors.

**Methods:**

For this cross-sectional study, 2530 students were chosen with stratified cluster sampling among 20,493 high school students studying in Bornova district of Izmir, Turkey, among whom 2240 (88.5%) participated. Risk perceptions and opinions were questioned with a 5-point Likert scale for 24 statements grouped under four dimensions. The mean responses to the four dimensions were categorized as <3.5 (low) and ≥3.5 (high) and the determinants were analyzed with logistic regression.

**Results:**

Mean risk perception scores for the mobile phone, base station, trust to authority and incivility dimensions were 3.69 ± 0.89, 4.34 ± 0.78, 3.77 ± 0.93, 3.16 ± 0.93 and the prevalence of high risk perception was 65.1%, 86.7%, 66.2%, 39.7%, respectively. In the mobile phone dimension; students attending industrial technical high school had lower risk perceptions while female students, lower mothers’ education groups and students not using mobile phones (OR = 2.82, 95% CI = 1.80-4.40) had higher risk perceptions. In the base station dimension girls had higher risk perceptions (OR = 1.68, 95% CI = 1.20-2.37). Girls and students attending industrial technical high school had significantly lower risk perception however 11-12th grade group perceived the risk higher (OR = 1.45 95% CI = 1.15-1.84) in the trust to authority dimension. For the incivility dimension, female students (OR = 1.44, 95% CI = 1.14-1.82), illiterate/only literate mothers (OR = 1.79, 95% CI = 1.04-2.75) and students not using mobile phones (OR = 2.50, 95% CI = 1.62-3.87) perceived higher risk.

**Conclusions:**

Understanding the effects of these determinants might aid in developing more effective educational interventions to specific subgroups on this topic. As debates on the health consequences of electromagnetic fields continue, it would be cautious to approach this issue with a preventive perspective. Efforts should be made to equalize the varying level of knowledge and to ensure that students are informed accurately.

## Background

Mobile phone use has become widespread among children and adolescents, with surveys finding 76% mobile phone ownership in Hungary, 79% mobile phone access in Sweden and 94% ownership in Germany [1-3]. The ratio of adolescent users is estimated to have further increased [4] and politicians, public health officials and the scientific community show an increasing interest in the relation between children and adolescents’ health and radiofrequency radiation (RFR) exposure [5].

A recent review on epidemiological studies shows no or only limited evidence on the effects of RFR on the incidence of brain tumors and leukemia in children as well as cognitive and other brain functions in children. The same review however emphasizes that the available literature cannot rule out adverse health effects of RFR due to the impossibility to prove a non-effect and due to the remaining knowledge gaps [5]. Several of the Interphone studies have found increased risks of glioma especially on the ipsilateral side after 10 years of exposure [6-9], however the pooled analysis of Interphone studies [10] concluded that “no increase in risk of glioma or meningioma was observed with use of mobile phones. The possible effects of long-term heavy use of mobile phones require further investigation”. As carcinogenesis might need a longer period like 20–30 years, the currently negative findings do not implicate the absence of risk and International Agency for Research on Cancer (IARC) has recently classified mobile phone-related RFRs as group 2B carcinogen, i.e. possibly carcinogenic to humans (limited evidence of carcinogenity in humans and less than sufficient evidence in experimental animals) based on an increased risk for glioma associated with wireless phone use [11]. Children and adolescents start to use mobile phones at an earlier age compared to adults, in a period when the plasticity of their brain continues [12]. The exposure to RFR still continues to have a high research priority according to the new World Health Organization (WHO) research agenda [13,14]. Indeed, a recent study has found an increased risk of glioma among people who had started to use mobile phones under the age of 20 [15].

With the recent possibility of internet access through mobile phones, and with the several other options of communication that mobile phones offer, adolescents who enter in a more extrovert period of their lives might prioritize these communication opportunities more than their health. Compared to adults, teenagers perceived lower risk in experimental and occasional involvement in health-threatening activities [16]. Adolescents’ participation in risky behavior is shown to be linked to their risk perceptions [17] and these behaviors may persist throughout life in the form of habits [18]. Therefore it is crucial to study adolescents’ risk perceptions. However, studies on adolescents’ risk perceptions of mobile phones or base stations are very scarce.

Studies on risk perception characterize and evaluate the opinions of people on hazardous activities and technologies [19]. The psychometric approach focuses on the identification of factors determining public perception of different hazards [20,21]. The cultural approach focuses on the effects of cultural adherence and social learning [22]. Worldviews are also considered as orienting dispositions guiding people’s decisions [23]. However these models were criticized for not providing answers to all the questions regarding perceived risk [24,25]. Several studies indicate that there are substantial individual differences in risk perception [26,27]. Risk perception strongly relies on age, gender, education and culture [28,29]. While such general factors are known as determinants of perceived risk, much less is known about the specific determinants of RFR risk perception [13] and socioeconomic determinants of risk perception have not been studied especially in developing countries like Turkey. Adolescents constitute approximately 20% of Turkey’s [30] population and qualitative and quantitative differences exist among the infrastructures, education systems and health services of the schools [31-33].

Our main study questions were; i. Are there differences in the risk perceptions of high school students? Do they trust the authorities and regulations regarding RFR exposure? How polite do they use mobile phones? ii. How do socioeconomic factors, demographic factors and the use of this technology relate to their risk perception, trust in authorities and attitudes of incivility while using this technology?

The present study aims to evaluate the risk perception on cell phone use and base stations, their trust to authorities and their opinions on incivility in using mobile phones and to assess the associated factors, among high school students in the Bornova district of Izmir.

## Methods

### Subjects

The data of this cross-sectional study were collected between 7 December 2009 and 15 April 2010 in Bornova district of Izmir. Located on the Aegean coast, Izmir is the third largest city of Turkey with 3.7 million population. Bornova is one of its metropolitan districts with 419,070 population size. There is a central high school entrance examination which enables students from different provinces of Turkey to enter a high school in any part of the country according to the points they get from the exam. Among districts of Izmir, Bornova is unique with its high school education infrastructure, having two famous high schools favored from all around the country and also two high-capacity industrial technical high schools. All high school students in Bornova (a total of 26 high schools and 20,493 students) comprised the target group of the survey. A sample size of 2530 was calculated with 50% prevalence (to maximize the sample size in the case of an unknown prevalence), 3% error, 95% confidence level, design effect 2 (to prevent clustering effect) and 20% non-response. Stratified cluster sampling was applied with stratification according to school size and classes as clusters. A total of 87 classes out of 704 have been chosen in a systematic random manner to reach this sample size, including all the students in the selected classrooms. The actual number of students registered to these 87 classes was 2466, and 2240 (90.8%) were present in the classroom during data collection and none of them refused to participate in the study.

### Instrument and scoring

A questionnaire in Turkish language was developed in accordance with previous studies conducted on the same topic [18,27] and distributed by the researchers to the students during class hours. A researcher and a teacher were present in the classroom while the students filled out the questionnaires.

#### Variables

**Figure 1:**
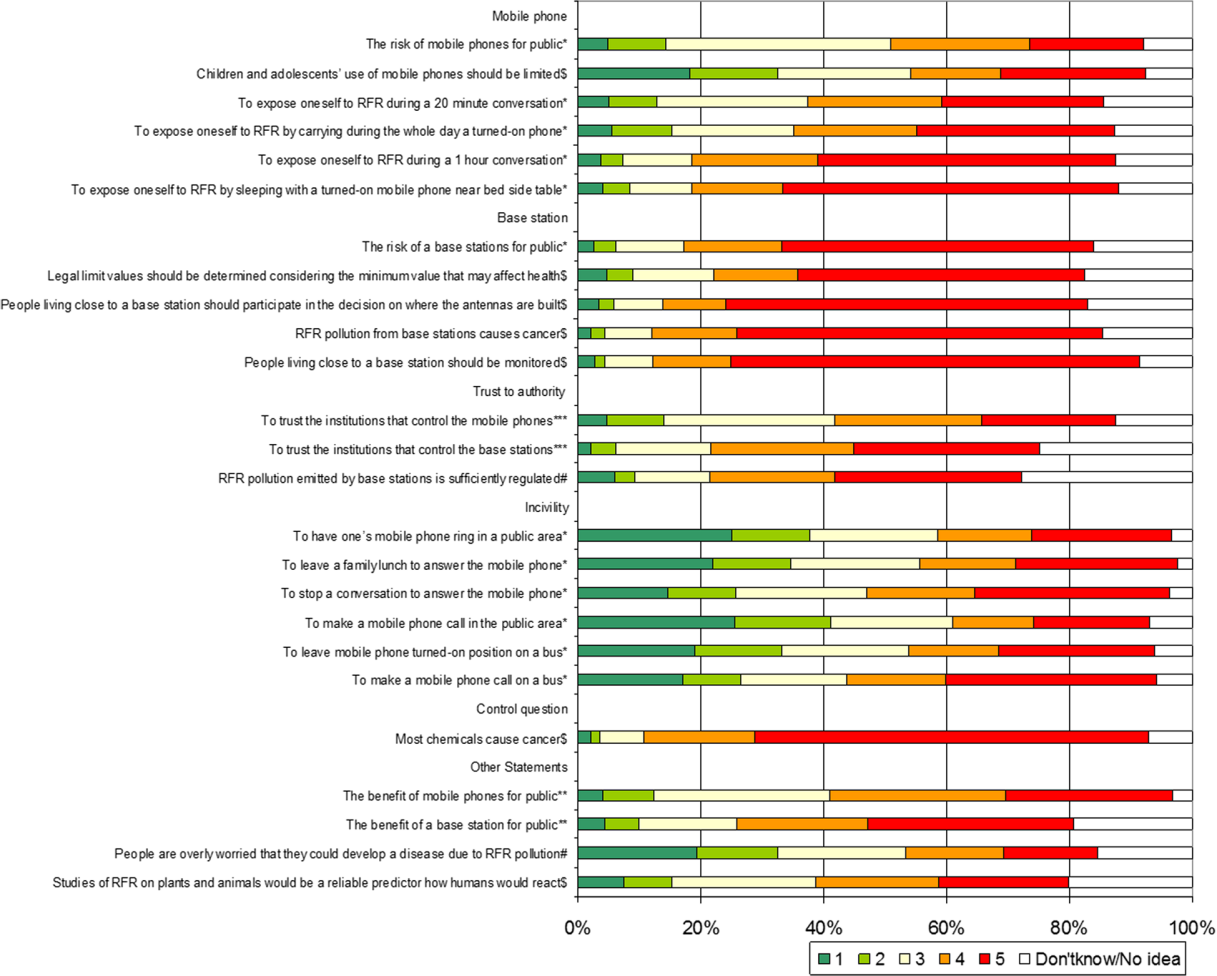
Risk perceptions and opinions of the students among mobile phone, base station, trust, incivility dimensions and other statements. * ‘1’ no risk; ‘5’ very high risk, ** ‘1’ very beneficial; ‘5’ no benefit, *** ‘1’ very high trust; ‘5’ no trust, $ ‘1’ no agreement; ‘5’ absolutely agree, # ‘1’ absolutely agree; ‘5’ no agreement.

Risk perceptions and opinions of students were questioned with a 5-point Likert scale for 24 statements and one control question on the carcinogenicity of chemicals. The students were not forced to express an opinion for each item, since a “have no idea” option was included along with the 5-point Likert scale. A principal axis factor analysis with oblique rotation was used on the 24 items and 20 of them were classified under four dimensions as mobile phone, base station, trust to authority and incivility. The Cronbach’s alpha coefficient of the overall 20 items was 0.78, and 0.79, 0.71, 0.58 and 0.67 for the four dimensions, respectively. The four items left out were on the benefits of this technology, whether people were overly worried about RFR and the generalizability of RFR studies conducted on plants and animals (Figure 1).

In the mobile phone dimension, five risk perception and one opinion statements were asked (‘1’ no risk, ‘5’ very high risk; ‘1’ no agreement, ‘5’ absolutely agree). In the base station dimension, one risk perception statement and four opinion statements were asked (‘1’ no risk, ‘5’ very high risk; ‘1’ no agreement, ‘5’ absolutely agree). In the trust to authority dimension, two statements evaluated the trust to regulatory institutions and one statement asked for the opinion on regulations (‘1’ very high trust, ‘5’ no trust; ‘1’ absolutely agree; ‘5’ no agreement). The incivility dimension included six statements about the social risks (‘1’ no risk, ‘5’ very high risk). Due to the “have no idea” option which gave no score, mean scores were calculated for each dimension instead of total scores. If a respondent replied “have no idea” to an item, his mean score for that dimension was calculated with the remaining items that he had scored. Based on the crude overall mean risk perception score throughout all the items in the questionnaire before the factor analysis and also based on previous studies [18], a cut-off level of 3.5 points was determined for the mean dimension scores and the students were grouped into two categories “high risk perception” and “low risk perception” according to this cut-off. So, the outcome variables were converted into dichotomous variables.

Gender, age, grade, income, mother’s education, social class, type of school and mobile phone utilization were the independent variables of the study. Age: Completed age was calculated by using the birth date and the date when the questionnaire was applied. Grade: Since 2005, high school education in Turkey lasts four years, after eight years of primary education. It was grouped as 9-10th and 11-12th grades. Income: The total household income was divided by the number of household members in order to obtain income per capita. Income per capita was grouped into five categories with 200 Turkish Lira (TL) intervals. Currencies were converted into Euros as of February 10, 2010, the mid of the data collection period. Mother’s education was grouped according to the educational achievements in Turkish national education system: completion of primary school (5 years), secondary school (3 years), high school (3 years) and university (at least 2 years). Mothers who had not completed primary school but could read and write (only literate) and those who could not read or write (illiterate) were combined. Social class: Class position was determined according to the father’s, or, if not present or if not working, the mother’s occupation [34]. The categorization was based on the class scheme developed by Boratav [35]. Type of school: The 26 high schools were classified according to five main school categories (standard high school, trade high school, technical high school for girls, industrial technical high school and Anatolian/science/private high school). Students can enter standard high schools without passing the central examination. Trade, girls’ technical and industrial technical schools are vocational high schools. Most of their programs also do not require central examination for entrance, while they have very few special programs requiring a reasonable score in the central exam. Anatolian high schools and private schools predominantly teach in foreign languages. Science high schools have a curriculum mainly based on science and research projects. Students need to have high scores to enter the Anatolian and Science high schools. Mobile phone utilization: Students were asked if they used a mobile phone (yes/no).

### Data analysis

Spearman’s correlation analysis was performed to see whether there was a correlation between chemical risk perception and RFR risk dimensions. The mean scores of the four dimensions were compared with repeated measures ANOVA. The relation of risk perception with the independent variables was determined with *X*^2^ tests. The variables in relation with the dependent variables in these hypothesis tests were included in the logistic regression analysis. Anatolian/science/private high school, boys, 9-10th grade, highest income (601 TL+), white collar/manager, highest education (13 + years), users of mobile phone were the reference categories. Results were presented as odds ratios (OR) with 95% confidence intervals (CI). Analyses were made separately for mobile phone, base station, trust to authority and incivility dimensions. For each dimension, only the variables found statistically significant in bivariate analyses are included in multivariate analysis. We saw the independent effect of each variable in the first step (Crude) and then controlled for all the variables in the equation (Adjusted). A value of p < 0.05 (2-sided) was considered statistically significant. Statistical analysis was performed using the software SPSS version 16.0 (SPSS Inc. Chicago, IL).

## Results

Among the participating 2240 students, 54.6% were male. The mean age of the respondents was 16.5 ± 1.3 years (±SD; standard deviation) (min-max: 14–21). Table 1 shows the detailed distribution of the surveyed population according to socio-demographic characteristics.

**Table 1:** The distribution of the surveyed population according to socio-demographic characteristics (n = 2240)

**Characteristic**	**n**	**%**
**Sex**		
Female	1150	48.4
Male	1078	54.6
**Age**		
≤15	572	26.8
16	593	27.8
17	457	21.4
≥18	514	24.1
**Grade**		
9-10	1299	60.4
11-12	851	39.5
**Income per capita (Euros)**		
Lowest (0–200 TL) (0–97 Euros)	479	30.7
Lowest middle (201–400 TL) (98–193 Euros)	555	35.6
Upper middle (401–600 TL) (194–290 Euros)	216	13.8
Highest (601 TL +) (291 Euros and over)	311	19.9
**Social class**		
Blue collar/Unemployed	956	46.9
Self Employed	444	21.8
Employer	170	8.3
White collar/Manager	468	23.0
**Mother’s Education**		
Illiterate/Only literate	139	6.4
Primary (5 years)	908	41.7
Secondary (8 years)	370	17.0
High school (11 years)	426	19.6
University (13+ years)	332	15.3
**School Type**		
Trade high school	285	13.3
Technical high school for girls	212	9.9
Standard high school	558	26.0
Industrial technical high school	489	22.7
Anatolian/Science/Private high school	606	28.2
**Mobile phone utilization**		
Yes	2005	92.0
No	175	8.0

The ratio of students using mobile phones increased with increasing grade, income per capita, father’s social class and mother’s education (p trend < 0.05).

Figure 1 presents the distributions of the students according to their responses to the 24 statements on risk perceptions and opinions, grouped under the four dimensions.

The mean risk perception scores and ± SD for the mobile phone, base station, trust to authority and incivility dimensions were 3.69 ± 0.89, 4.34 ± 0.78, 3.77 ± 0.93, 3.16 ± 0.93, respectively, all of them significantly different from each other (p < 0.001). The percentage of students who perceived a high risk (>3.5) for the mobile phone, base station, trust to authority and incivility dimensions were 65.1%, 86.7%, 66.2%, 39.7%, respectively.

Chemical risk perception was weakly correlated with mobile phone and incivility dimensions, the correlation coefficients being 0.20 and 0.07, respectively, moderately correlated with base station (r = 0.32) and not correlated with trust to authority dimensions (r = 0.00).

Table 2 presents the prevalence and odds ratios of high risk perception in mobile phone and base station risk dimensions in relation to socio-demographic variables.

**Table 2:** The prevalence and odds ratios of high risk perception in mobile phone and base station dimensions in relation to socio-demographic variables

		**Mobile phone**			**Base station**	
	**High risk perception (%)**	**Crude odds ratio (95% CI)**	**Adjusted# odds ratio (95% CI)**	**High risk perception (%)**	**Crude odds ratio (95% CI)**	**Adjusted# odds ratio (95% CI)**
**Sex**						
Female	71.2	1.59 (1.29–1.97)**	1.60 (1.32–.1.95)**	89.9	1.62 (1.19–2.20)*	1.68 (1.20–2.37)*
Male (ref)	60.8	1.00	1.00	84.5	1.00	1.00
**Grade**						
11-12	67.8	1.16 (0.94–1.45)	-	89.6	1.42 (1.03–1.96)*	1.38 (0.99–1.92)
9-10 (ref)	64.4	1.00	-	85.9	1.00	1.00
**Income per capita**						
Lowest (0–200 TL)	69.3	1.28 (0.95–1.74)	-	83.2	0.59 (0.38–0.91)*	0.74 (0.44–1.25)
Lowest middle (201–400 TL)	66.9	1.15 (0.86–1.54)	-	87.3	0.83 (0.53–1.29)	1.04 (0.62–1.72)
Upper middle (401–600 TL)	59.8	0.85 (0.59–1.21)	-	92.4	1.46 (0.78–2.72)	1.59 (0.84–3.03)
Highest (601 TL +) (ref)	63.8	1.00	-	89.3	1.00	1.00
**Social class**						
Blue collar/Unemployed	67.4	1.20 (0.92–1.57)	-	85.2	0.87 (0.60–1.26)	-
Self Employed	66.2	1.14 (0.82–1.57)	-	89.2	1.24 (0.77–2.01)	-
Employer	62.8	0.98 (0.61–1.57)	-	92.4	1.82 (0.79–4.19)	-
White collar/Manager(ref)	63.3	1.00	-	87.0	1.00	-
**Mother’s Education**						
Illiterate/Only literate	75.5	2.33 (1.36–3.99)*	1.77 (1.01–2.86)*	92.6	1.39 (0.58–3.37)	-
Primary (5 years)	68.1	1.61 (1.18–2.19)*	1.66 (1.20–2.30)*	85.9	0.69 (0.42–1.11)	-
Secondary (8 years)	67.0	1.53 (1.07–2.19)*	1.64 (1.19–2.27)*	84.2	0.60 (0.35–1.03)	-
High school (11 years)	64.2	1.35 (0.94–1.94)	1.36 (0.95–1.94)	88.7	0.88 (0.50–1.55)	-
University (13+ years) (ref)	57.0	1.00	1.00	89.9	1.00	1.00
**School Type**						
Trade high school	71.9	1.43 (0.99–1.79)	0.89 (0.62–1.27)	84.8	0.56 (0.33–0.92)*	0.58 (0.32–1.04)
Technical high school for girls	69.9	1.33 (0.98–1.73)	0.81 (0.55–1.18)	87.2	0.68 (0.38–1.21)	0.66 (0.34–1.25)
Standard high school	66.5	1.13 (0.77–1.67)	0.88 (0.66–1.17)	88.8	0.79 (0.49–1.26)	0.83 (0.49–1.39)
Industrial technical high school	59.5	0.75 (0.59–0.96)*	0.61 (0.45–0.83)*	83.6	0.51 (0.33–0.78)*	0.66 (0.40–1.16)
Anatolian/Science/Private high school (ref)	63.7	1.00	1.00	91.0	1.00	1.00
**Mobile phone utilization**						
No	85.8	3.37 (2.02–5.62)**	2.82 (1.80-4.40)*	86.4	0.93 (0.55-1.59)	-
Yes (ref)	64.2	1.00	1.00	87.2	1.00	1.00

#: adjusted for all variables present in the table for that outcome variable *p < 0.05, ** p < 0.001.

–: variables that were not found significant in univariate analysis so not included in the model.

In the mobile phone dimension, girls (OR = 1.59, 95% CI = 1.29-1.97) and students not using mobile phones (OR = 3.37, 95% CI = 2.02-5.62) had statistically significantly higher risk perceptions. An increase in risk perception of the students was observed with decreasing mother’s educational level (p for trend = 0.003). The ORs and 95% CI for the secondary, primary and illiterate/only literate groups were 1.53 (1.07–2.19), 1.61 (1.18-2.19) and 2.33 (1.36-3.99), respectively. Students attending industrial technical high school had lower risk perceptions with OR = 0.75 (95% CI = 0.59-0.96). After the adjustment, these four variables remained statistically significant. The size of the risk was nearly the same for girls OR = 1.60 (95% CI = 1.32–1.95). The same gradient remained for mother’s education variable with small differences in ORs. The highest risk was found for the students not using mobile phones with OR = 2.82 (95% CI = 1.80-4.40) in this dimension (Table 2).

In the univariate analyses, girls and 11-12th grade students perceived higher base station risk with OR = 1.62 (95% CI = 1.19-2.20) and OR = 1.42 (95% CI = 1.03-1.96). Industrial technical high school and trade high school students had statistically significantly lower risk perceptions in the crude analysis (OR = 0.51, 95% CI = 0.33-0.78 and OR = 0.56, 95% CI = 0.33-0.92). After the adjustment, the significances disappeared except for gender, the OR for girls becoming 1.68 (95% CI = 1.20-2.37) (Table 2).

Table 3 presents the prevalence and odds ratios for high risk perception in trust to authority and incivility dimensions in relation to socio-demographic variables, school type, grade and cell phone utilization.

**Table 3:** The prevalence rates and odds ratios for trust and incivility dimensions of high school students in relation to the independent variables

		**Trust**			**Incivility**	
	**High risk perception (%)**	**Crude odds ratio (95% CI)**	**Adjusted# odds ratio (95% CI)**	**High risk perception (%)**	**Crude odds ratio (95% CI)**	**Adjusted# odds ratio (95% CI)**
**Sex**						
Female	63.5	0.82 (0.66–0.97)*	0.74 (0.58–0.95)*	44.0	1.40 (1.14–1.72)*	1.44 (1.14–1.82)*
Male (ref)	67.9	1.00	1.00	35.9	1.00	1.00
**Grade**						
11–12	69.7	1.34 (1.07–1.67)*	1.45 (1.15–1.84)*	40.6	1.05 (0.85–1.30)	-
9–10 (ref)	63.2	1.00	1.00	39.4	1.00	-
**Income per capita**						
Lowest (0–200 TL)	63.6	0.67 (0.49–0.92)*	1.19 (0.76–1.87)	46.4	1.66 (1.22–2.24)*	0.80 (0.51–1.23)
Lowest middle (201–400 TL)	60.8	0.60 (0.44–0.80)*	0.90 (0.60–1.40)	41.0	1.34 (1.00–1.79)*	0.77 (0.51–1.15)
Upper middle (401–600 TL)	73.2	1.05 (0.71–1.56)	1.40 (0.90–2.18)	32.4	0.92 (0.64–1.34)	0.67 (0.44–1.03)
Highest (601 TL +) (ref)	72.3	1.00	1.00	34.2	1.00	1.00
**Social class**						
Blue collar/Unemployed	60.9	0.59 (0.45–0.78)**	0.76 (0.54–1.07)	43.1	1.48 (1.14–1.94)*	1.18 (0.73–1.90)
Self Employed	66.8	0.76 (0.54–1.07)	0.84 (0.50–1.40)	39.8	1.29 (0.82–2.05)	0.99 (0.69–1.41)
Employer	68.1	0.81 (0.49–1.32)	0.89 (0.62–1.29)	38.3	1.21 (0.88–1.68)	1.04 (0.75–1.44)
White collar/Manager(ref)	72.5	1.00	1.00	33.8	1.00	1.00
**Mother’s Education**						
Illiterate/Only literate	65.6	0.64 (0.38–1.07)	0.88 (0.40–1.54)	46.7	2.34 (1.41–3.86)**	1.79 (1.04–2.75)*
Primary (5 years)	62.4	0.55 (0.39–0.78)*	0.83 (0.50–1.37)	44.9	2.17 (1.56–3.02)**	1.69 (0.93–2.46)
Secondary (8 years)	62.5	0.56 (0.38–0.82)*	0.84 (0.50–1.41)	39.5	1.74 (1.19–2.54)*	1.38 (0.84–2.29)
High school (11 years)	68.4	0.72 (0.49–1.06)	0.92 (0.58–1.47)	37.9	1.62 (1.12–2.36)*	1.44 (0.92–2.25)
University (13+ years) (ref)	75.0	1.00	1.00	27.3	1.00	1.00
**School Type**						
Trade high school	61.7	0.55 (0.39–0.79)*	0.69 (0.44–1.09)	44.3	1.85 (1.31–2.61)**	1.49 (0.91–2.37)
Technical high school for girls	64.7	0.63 (0.43–0.94)	0.85 (0.53–1.38)	46.8	2.05 (1.41–2.99)**	1.57 (0.99–2.48)
Standard high school	65.8	0.66 (0.49–0.90)	0.86 (0.59–1.26)	43.1	1.76 (1.31–2.37)**	1.46 (0.89–2.22)
Industrial technical high school	59.9	0.49 (0.37–0.67)**	0.57 (0.38–0.85)*	42.0	1.68 (1.25–2.26)**	1.47 (0.89–2.23)
Anatolian/Science/Private high school (ref)	74.4	1.00	1.00	30.1	1.00	1.00
**Mobile phone utilization**						
No	61.3	0.81 (0.56–1.19)	-	60.2	2.43 (1.67–3.54)**	2.50 (1.62–3.87)**
Yes (ref)	66.0	1.00	-	38.3	1.00	1.00

#: adjusted for all variables present in the table for that outcome variable *p < 0.05, ** p < 0.001.

–: variables that were not found significant in univariate analysis so not included in the model.

In the trust to authority dimension, the 11-12th grade group had higher risk perception which meant lower trust to authority (OR = 1.34, 95% CI = 1.07-1.67). Girls, lowest and lowest middle income groups, blue collar or unemployed group, mothers completing primary and secondary school, students attending trade high school and industrial technical high school had statistically significantly lower risk perception which meant higher trust to authority. After the adjustment, the same patterns were observed for girls and 11-12th grade group (OR = 0.74, 95% CI = 0.58-0.95, OR = 1.45 95% CI = 1.15-1.84) while income per capita, social class, mother’s education and trade high school lost significance and did not remain in the adjusted analysis. Students attending industrial technical high school showed significantly higher trust to authority, with OR = 0.57 (95% CI = 0.38-0.85) (Table 3).

For the incivility dimension, in the crude analysis, female students, lowest income groups, blue collar, unemployed and self employed groups and students not using mobile phones had statistically significantly higher risk perceptions. Additionally, a statistically significant increase in risk perception of the students was observed with decreasing mother’s educational level (p for trend = 0.001). Students attending technical high school for girls, trade high school, industrial technical high school and standard high school perceived incivility risks statistically significantly higher than the reference group. After the adjustment female students, illiterate and only literate mothers group remained statistically significant with OR = 1.44, (95% CI = 1.14-1.82) and OR = 1.79 (95% CI = 1.04-2.75), respectively. Students not using mobile phones also had statistically significantly higher risk perceptions with an increase in the odds ratio to 2.50 (95% CI = 1.62-3.87) (Table 3).

## Discussion

Risk perception was highest in the base station dimension while it was lowest for the incivility dimension. Gender was associated with all dimensions of RFR-related risk perception, with opposite pattern observed for the trust dimension. Students attending industrial technical high school had a low risk perception in the mobile phone and trust dimensions. Students not using mobile phones had a higher risk perception about mobile phones and incivility. Adolescents in the 11-12th grades had a higher risk perception in the trust dimension. A gradual increase was observed in mobile phone risk perception with decreasing mother’s educational level. Students having illiterate and only literate mothers perceived the social risks lowest in the incivility dimension.

The statement “children and adolescents’ use of mobile phones should be limited” was the least supported idea in the mobile phone risk perception dimension. This might show that despite the perception of some risks, the students are against a limitation of their freedom to use mobile phones, which might be related with the fact that mobile phone operators frequently use the image of freedom in their advertisements. To expose oneself to RFR when sleeping with a turned-on mobile phone near the bed side was the most highly perceived risk statement among the mobile phone dimension items, even though the exposure is negligible compared talking on the phone. This statement was followed closely by exposing oneself to RFR during a one hour conversation.

The highest risk perceptions are about statements pertaining to base stations. Other studies have also revealed a considerable public concern that living in the vicinity of a mobile phone base station has adverse effects on health [36]. Although exposures from base stations are very low with respect to exposures to mobile phones, the reason of this high perception might be related to the continuous exposure from base stations or the effect of a widespread belief on adverse health effects of these technologies which was popularized by the opposing groups. The statements on the necessity of follow-up for people living close to base stations, participation into decisions on the setting up of base stations and cancer risk from base stations found higher support than a nationwide study from Germany [36]. This might be related to information provided by the media or NGOs. The statement receiving the highest support was that the legal limit values should be determined according to the lowest level affecting human health. Legal limit values are the most important bases to rely on to protect from possible health risks. The lack of clarity on the long-term effects renders the current limit values questionable. More than half of the students either think that the issue is exaggerated or they are undecided. This might be a consequence of uncertainty due to contradictory information received from different sources. It was interesting to note that the base station dimension had more correlation with chemical risk perception than the other dimensions, though the correlation coefficient was smaller than Siegrist’s [27] who concluded that people concerned about chemical substances were also concerned about RFR.

In the trust to authority dimension we observed the highest “have no idea” response to the statement whether RFR pollution emitted by base stations was sufficiently regulated. A similar level of mistrust to authorities was observed among the items in this dimension. While mistrust to authority could be related to the period of adolescence *per se*, the finding that young people’s levels of trust related to the stability of democracy in different countries could also partly explain this low level of trust [37].

Students perceive much lower risks for the incivility dimension in comparison to other countries [18]. This is probably related to cultural issues. In contrast to European countries where answering the phone during a face-to-face communication with someone else is considered impolite, not answering the phone or answering the phone lately are considered as impoliteness (towards the caller) in Turkey. Riding a bus with the mobile phone turned-on was the statement that had the highest perceived risk in this dimension. Mobile phones were once prohibited on many buses in Turkey due to the risk of interference with the braking systems, so the high scores could more be related to security issues instead of politeness.

Gender differences in risk perception have been shown previously [27,38]. In the present study girls seem to be more concerned than boys in mobile phone, base station and incivility dimensions. Punamaki indicated that girls were also vulnerable to the negative consequences of intensive mobile phone usage, as it was associated with perceived health complaints [39]. Female students perceived lower risk in the trust to authority dimension which means that girls trust to regulatory authorities more than boys. This point might be explained by the patriarchal nature of their society [40].

Students in the 11-12th grades trust to regulatory authorities less than the 9-10th grades. The 11-12th grades might have more information or awareness about the working process of the regulatory authorities or it might be related to their period of adolescence. A study has found that risk perceptions are strongly associated with general concern and stress [41], thus the stress of the upcoming university entrance exam might be increasing their risk perceptions.

Adolescents with less educated mothers perceive mobile phone and incivility risks higher in this study. Consistently, Blettner’s study has found similar associations with income and education [36]. For the mobile phone dimension, the high risk perceptions of adolescents with less educated mothers might be related to a rationalization of their lower access to this technology.

School type was associated with mobile phone and trust to authority dimensions. Industrial technical high school students showed statistically significantly lower risk perception in these dimensions. There could be several explanations for this difference: They might be given training on the production or reparation of various devices emitting RFR and they might be underestimating the risks associated with such devices as they might be closely working with them, or they might perceive other RFR sources such as transformers or high power lines more risky or the presence of more apparent risks in their working environments might lead to a lower perceived RFR risk. Overall, the curricula applied in different types of school, the teachers’ differing attitudes and the difference of policies applied at different schools to control the use of mobile phones at school and during the classes might also have altered the perceived risks of students. For example one of the participating schools provided lockable cabinets for the students to lock their mobile phones upon arrival to school, or it was strictly forbidden to bring mobile phones to school in another, while students could keep their mobile phones turned on in silent mode during the classes in another school.

Mobile phone utilization was an important predictor of risk perception for two of the dimensions, mobile phone and incivility. Base station and trust dimensions were independent of this variable. Students who did not use mobile phones perceived the risks at least two and a half-fold higher for mobile phone and incivility dimensions. These could either result from a rationalization of users who tended to underestimate the risk, or to a rationalization by non-users that there is an advantage by not using mobile phones. Although insignificant, cell phone users showed a higher risk perception than non-users for base stations, though their cell phone risk perception was lower. This might be explained by the unknown and dread risk concepts described by Slovic. The perception of base station risks as uncontrollable and involuntary might have caused their perception independent of their cell phone utilization [20]. The significant difference between the mean scores of these two dimensions supports this idea. In the early 1990s, mobile phone was considered to be a prestigious object as a symbol of financial independency [3]. The fact that socio-economically disadvantaged students had less access to this technology rules out the other option of a deliberate decision by the parents/students not to use mobile phones due to health risks, indicating that the chicken or the egg dilemma is not present in this association. The inverse relationship between mobile phone use/ownership and associated risks are also observed in other studies [18,27].

### Strengths and limitations

Although generalizability of the study findings may be considered limited because the study subjects are sampled from a local area, information generated from the data can be of help in developing risk communication strategies on potential RFR risk. Additionally, the same school types are widespread all over the country and Bornova’s population size is greater than most of the districts and even most of the provinces in Turkey. The strengths of this study are its large sample size and good coverage rate, and its opportunity to compare between different school types and many socioeconomic characteristics. In this study not only risk perceptions are assessed, but also items related to trust in actors involved in risk management, the perceived quality of risk management and incivility issues related to the use of this technology.

A limitation is that schooling is not mandatory for this age group, so the adolescents not attending high schools are not covered. The response rate of the variable income per capita was low (69.7%). This could be expected since students might not be well informed about the actual monthly income of their parents. Another limitation could be in the self–reports of adolescents which might cause some bias.

## Conclusions

In conclusion, risk perceptions about mobile phones and base stations differ primarily according to the sex and partly according to mobile phone utilization, school type and mother’s educational level. Risk perception was highest in the base station dimension, probably due to a continuous exposure that people cannot have a control on, while it was lowest for the incivility dimension which is a reflection of the different cultural approach in Turkey. An understanding of the effects of socioeconomic and mobile phone utilization characteristics might aid in developing more effective risk communication to specific subgroups. While debates on the health consequences of RFR continue, it would be cautious to approach this issue with a preventive perspective. In this view, efforts should be made to improve and standardize the varying level of knowledge in different schools and to ensure that students are informed accurately. Health education on the correct use of this technology should include appropriate messages. Documents including standard recommendations could be prepared by experts assigned by the Ministry of Education and used during these educational activities.

### Human subjects approval statement

Permissions to conduct this study were obtained from the National Education Directorate in Bornova and the Provincial Directorate of National Education. Ethical approval was obtained from Izmir Number 1 Ethics Committee on September 7, 2009 with the decision number 09-9/8.

## Abbreviations

RFR: Radiofrequency radiation; IARC: International agency for research on cancer; WHO: World health organization; TL: Turkish lira.

## Competing interests

The authors declare that they have no competing interests.

## Authors’ contributions

HH, RD and AOK have made contributions to conception and design and acquisition of data, HH and RD have made analysis and interpretation of data; HH and RD have been involved in drafting the manuscript and revising, HH, RD and AOK have given final approval of the version to be published. All authors have participated sufficiently in the work to take public responsibility for appropriate portions of the content.
